# Scaling-up Fermentation of *Pichia pastoris* to demonstration-scale using new methanol-feeding strategy and increased air pressure instead of pure oxygen supplement

**DOI:** 10.1038/srep18439

**Published:** 2016-01-21

**Authors:** Wan-Cang Liu, Ting Gong, Qing-Hua Wang, Xiao Liang, Jing-Jing Chen, Ping Zhu

**Affiliations:** 1 State Key Laboratory of Bioactive Substance and Function of Natural Medicines; Institute of Materia Medica, Chinese Academy of Medical Sciences & Peking Union Medical College, 1 Xian Nong Tan Street, Beijing 100050, P.R. China; 2 Key Laboratory of Biosynthesis of Natural Products of National Health and Family Planning Commission, Institute of Materia Medica, Chinese Academy of Medical Sciences & Peking Union Medical College, 1 Xian Nong Tan Street, Beijing 100050, P.R. China

## Abstract

Scaling-up of high-cell-density fermentation (HCDF) of *Pichia pastoris* from the lab or pilot scale to the demonstration scale possesses great significance because the latter is the final technological hurdle in the decision to go commercial. However, related investigations have rarely been reported. In this paper, we study the scaling-up processes of a recombinant *P. pastoris* from the pilot (10 to 100-L) to the demonstration (1,000-L) scales, which can be used to convert 7-*β*-xylosyl-10-deacetyltaxol into 10-deacetyltaxol by the *β*-xylosidase for semi-synthesis of Taxol. We demonstrated that a pure oxygen supplement can be omitted from the HCDF if the super atmospheric pressure was increased from 0.05 to 0.10 ± 0.05 MPa, and we developed a new methanol feeding biomass*-stat* strategy (0.035 mL/g/h) with 1% dissolved oxygen and 100 g/L initial induction biomass (dry cell weight). The scaling-up was reproducible, and the best results were obtained from the 1,000-L scale, featuring a shorter induction time and the highest enzyme activities and productions, respectively. The specific growth and specific production rates were also determined. This study lays a solid foundation for the commercial preparation of 10-deacetyltaxol through the recombinant yeast. It also provides a successful paradigm for scaling-up HCDF of *P. pastoris* to the demonstration scale.

High-cell-density fermentation (HCDF) of recombinant *Pichia pastoris* has attracted significant attention in recent years. It is easy for *P. pastoris* to achieve ultra-high cell densities (>100 g/L dry cell weight, DCW or >400 g/L wet cell weight, WCW) in a simple and low-cost base salt medium^1^, which is more ideal for in fermenters than other expression systems. Meanwhile, *P. pastoris* can utilize the highly efficient alcohol oxidase 1 gene (*AOX1*) promoter which is induced by methanol for high-level expression of foreign genes. *P. pastoris* is usually cultured at 28 ~ 30 °C and pH5 ~ 6^2^. Under the proper culture temperature and pH conditions, sufficient oxygenation, or dissolved oxygen (DO) level, and methanol feeding strategy play decisive roles in both biomass production and heterologous protein expression. Since oxygen demand far exceeds the oxygen transfer capacity of conventional bioreactors such as stirred tanks, oxygen supply in aerobic HCDF processes remains a challenging task, which means DO is a constraint factor^3^,^4^,^5^. Increasing the DO concentration in equilibrium with the gas phase can be achieved by either using inlet air with a higher oxygen concentration (through the input of pure oxygen) or increasing total air pressure in the fermenter. Mixing pure oxygen into the inlet air flow is effective in small-scale fermentation^6^,^7^,^8^,^9^. However, in large-scale fermentation, supplying and handling pure oxygen can be highly expensive and dangerous. Comparatively, a more practical and economical strategy is to increase jar air pressure, especially for large-scale HCDF (a special tank is sometimes needed to tolerate the higher pressure). This strategy has been applied in certain aerobic microbial fermentations, such as *Escherichia coli*^10^,^11^, *Azotobacter vinelandii*^12^, *Corynebacterium glutamicum*^11^, *Ochromonas malhamensis*^10^, *Arxula adeninivorans*^11^, *Yarrowia lipolytica*^13^,^14^,^15^, *Kluyveromyces marxianus*^16^,^17^, *Saccharomyces cerevisiae*^18^,^19^ and *P. pastoris*^20^,^21^,^22^,^23^. Charoenrat *et al.*^21^ reported that increasing air pressure from 1.2 to 1.9 bar increased the oxygen uptake rate (OUR) of *P. pastoris* by 59%, and the heterologous *β*-glucosidase yield increased by 50% in the HCDF of the 10-L scale with ~120 g/L DCW. This may be the largest scale on which the strategy of increasing jar air pressure for the HCDF of *P. pastoris* has been adopted.

The P_*AOX1*_-regulated fed-batch cultivation of *P. pastoris* is divided into three stages: the glycerol batch stage (S1), glycerol fed-batch stage (S2) and methanol fed-batch stage (S3). S1 and S2 are collectively referred to as the biomass production phase, as producing sufficient biomass is their main objective. S3 is referred to as the induction phase; during S3, methanol is added to the medium and the biosynthesis of the heterologous protein is initiated. The added methanol is used as both the P_*AOX1*_ inducer and sole carbon source. Different methanol feeding strategies have different effects on specific (cell) growth rate (μ), residual methanol concentration and protein expression level. The most common methanol feeding strategies are DO control (DO-*stat*), specific growth rate control (μ*-stat*), oxygen limited fed-batch (OLFB) and methanol non-limited fed-batch (MNLFB). Potvin *et al.*^24^ and Barrigón *et al.*^25^ have informatively reviewed these strategies. Briefly, DO and μ are nearly constantly sustained using the DO-*stat* and μ*-stat* strategies, respectively. The main disadvantage of the DO-*stat* strategy is low reproducibility. This drawback can be overcome by using the μ*-stat* strategy. The OLFB strategy minimizes oxygen requirements and, therefore, may improve the economic feasibility of the process. The MNLFB strategy involves constantly maintaining the methanol concentration during the entire methanol induction phase. However, methanol depletion or accumulation may occur. Using optimal methanol feeding strategies, the yields of heterologous proteins could reach as high as 22 g/L^26^ for intracellular production of the recombinant hydroxynitrile lyase and 14.8 g/L^27^ for extracellular production of the recombinant gelatins. Frankly, there are no universal methanol feeding strategies for the HCDF of *P. pastoris*. What is more, methodologies often need to be modified, or even created, to meet the requirements of producing individual heterologous proteins.

The glycoside hydrolase LXYL-P1-2 (GenBank accession: AET31459) was cloned from the fungus *Lentinula edodes*^28^. This enzyme can specifically remove the xylosyl group from 7-*β*-xylosyl-taxanes, which are the by-products of the anti-tumor drug Taxol isolated from yew plants (*Taxus* species), thus forming the corresponding 7-*β*-hydroxyl-taxanes. 7-*β*-xylosyl-10-deacetyltaxol (XDT) is a type of 7-*β*-xylosyl-taxane, which contains dozens of times more natural content than Taxol. The hydrolytic product of XDT, 10-deacetyltaxol (DT), can be acetylated into Taxol. In order to produce DT from XDT on a massive scale using the enzyme LXYL-P1-2, we engineered a *P. pastoris* (designed as GS115-3.5K-P1-2) that harbored the *Lxyl-p1-2* gene (GenBank accession: JN167171) (intracellular expression) and carried out HCDF on the 10-L to 200-L scales^29^,^30^. Using freeze-dried HCDF cells as the bio-catalyst, we completed 10-L reaction volume bioconversion^30^ with a production efficiency of ~11 g/L. Compared with low-cell-density shaking flask fermentation, the aforementioned HCDF exhibited much higher volumetric enzyme activity, however its biomass enzyme activity decreased drastically. During HCDF, a pure oxygen supplement is required, but this is not economical or practical for large-scale fermentation. In this study, we focused on eliminating pure oxygen supplement during HCDF by significantly increasing the jar air pressure from 0.05 MPa to ~0.10 MPa (0.10  ± 0.05 MPa) and improving biomass enzyme activity by significantly increasing the volumetric enzyme activity of the HCDF of *P. pastoris*. In the end, we scaled up HCDF from the pilot scales of 10-L and 100-L to the demonstration/commercial scale of 1,000-L.

## Results

### Elimination of Pure Oxygen Supplement by Increasing Air Pressure

*Pichia* requires oxygen to metabolize glycerol and methanol. In order to maintain its high oxygen demand, a pure oxygen supplement is frequently required, apart from agitation and aeration, for high-cell-density fermentation (HCDF). Since the oxygen solubility of the liquid medium can be raised by increasing the total air pressure in the cultivation system, the desired DO can be maintained by keeping the jar air pressure higher in the absence of a pure oxygen supplement. The jar air pressure is normally set at ~0.05 MPa (super atmospheric pressure), but in this experiment it was set at 0.10 ± 0.05 MPa. The experiment with increased air pressure was designated as “Ip-Air”, meaning “increased air pressure without a pure oxygen supplement.” As a control, the normal experiment was designated as “Np-Ox”, meaning “normal air pressure (0.05 MPa) with a pure oxygen supplement.” The experiments were conducted in a 30-L scale fermenter using the DO-*stat* strategy.

**Figure 1:**
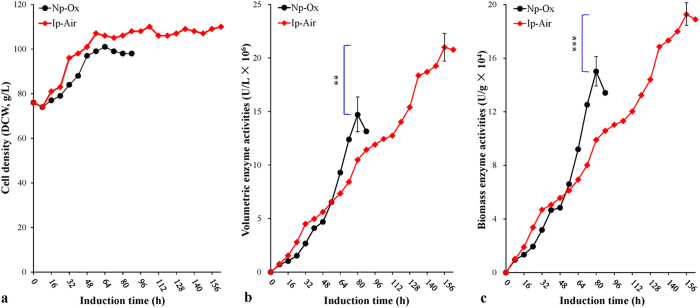
Time-course profiles of the HCDF under the two different conditions: Ip-Air (filled diamond) and Np-Ox (filled cycle). (**a**) Cell density profiles (DCW, g/L). (**b**) Volumetric enzyme activity profiles (U/L). (**c**) Biomass enzyme activity profiles (U/g). Data are means ± SD of three parallel measurements (*P < 0.05, **P < 0.01, ***P < 0.001). *Note*, Ip-Air: increasing air pressure without pure oxygen supplement; Np-Ox: normal air pressure (0.05 MPa) with pure oxygen supplement (control).

As shown in Fig. 1a, during the induction phase, the cell densities in both the Np-Ox and Ip-Air experiments increased steadily after a short delay. The biomass in the Np-Ox experiment reached its maximum value of 97.5 g/L (DCW) at 64 hours and then gradually fell. Similarly, the biomass in the Ip-Air experiment increased exponentially until 56 hours and then almost maintained a steady state with a maximum biomass of 107.5 g/L before the induction phase ended. Both specific growth rates (μ) increased gradually up to the average 0.0034 h^−1^ (Np-Ox) and 0.0040 h^−1^ (Ip-Air), respectively (Table 1). In the Np-Ox experiment (control), volumetric enzyme activity reached its maximum value of 1.5 × 10^7^ U/L at 80 hours. In the Ip-Air experiment, volumetric enzyme activity rose steadily until reaching the maximum value of 2.1 × 10^7^ U/L at 160 hours, which surpassed its control (*P* < 0.01) (Fig. 1b). Accordingly, maximum biomass enzyme activity (1.9 × 10^5^ U/g), maximum volumetric enzyme production (420 mg/L) and maximum biomass enzyme production (3.8 mg/g) in the Ip-Air experiment were obviously higher than those (1.5 × 10^5^ U/g, 294 mg/L and 3.0 mg/g) in the Np-Ox experiment (*P* < 0.001 for each comparison) (Fig. 1c; Table 1). The average specific enzyme production rate of the two experiments was nearly equivalent (0.023 mg/g/h *vs*. 0.022 mg/g/h, *P* > 0.05). These results indicate that a pure oxygen supplement can be omitted from the HCDF of *P. pastoris* if the air pressure is increased.

### Fermentation Using Biomass-*stat* Strategy

**Figure 2:**
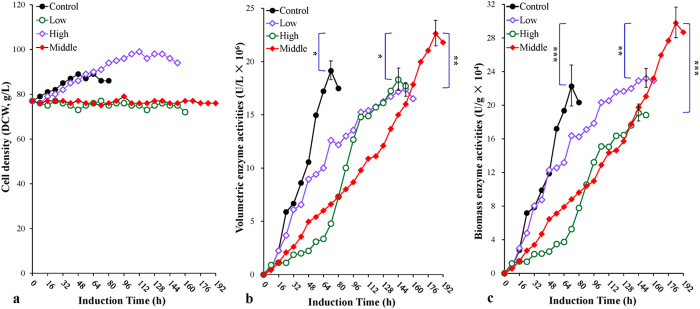
Time-course profiles of the biomass-*stat* fed-batch HCDF at low (open diamond, *v* = 0.025 mL/g/h), middle (filled diamond, *v* = 0.035 mL/g/h) and high (open cycle, *v* = 0.050 mL/g/h) methanol feeding rates and under Ip-Air condition. (**a**) Cell density profiles (DCW, g/L). (**b**) Volumetric enzyme activity profiles (U/L). (**c**) Biomass enzyme activity profiles (U/g). Data are means ± SD of three parallel measurements (*P < 0.05, **P < 0.01, ***P < 0.001). *Note*: the control was under Np-Ox condition with 10 mL/L/h methanol feeding.

Biomass-*stat*, a new methanol feeding strategy, was designed for the Ip-Air experiment to increase biomass enzyme activity as much as possible on the premise of maintaining the highest volumetric enzyme activity. HCDF was initiated in a 10-L scale fermenter. The optimal methanol feeding rate was determined to be 0.035 mL/g/h (middle feeding), compared with 0.025 mL/g/h (low feeding) and 0.050 mL/g/h (high feeding). As shown in Fig. 2a, during the induction phase, the biomass was maintained at the initial amount of 75 g/L at the low and middle feeding rates. For the high feeding rate, the biomass gradually increased from 75 g/L to nearly 100 g/L at 112 hours and then remained at this level or fell slightly. The control (Np-Ox, methanol feeding rate of 10 mL/L/h) shows a similar trend with the high feeding rate and the maximum biomass reached 95 g/L at 48 hours. For the middle feeding rate, maximum volumetric enzyme activity (Fig. 2b) reached 2.3 × 10^7^ U/L at 184 hours, the highest activity among the three feeding rates (the low feeding rate reached 1.7 × 10^7^ U/L at 152 hours and the high feeding rate reached 1.8 × 10^7^ U/L at 144 hours) (*P* < 0.01, *P* < 0.05 for each comparison) and surpassed that of the control (1.9 × 10^7^ U/L at 72 hours) (*P* < 0.05). However, the peak time was apparently delayed at all three feeding rates compared with the control, which requires improvement. Similarly, maximum biomass enzyme activity (3.0 × 10^5^ U/g), maximum volumetric enzyme production (452 mg/L) and maximum biomass enzyme production (6.0 mg/g) for the middle feeding rate exceeded those for the low feeding rate (2.3 × 10^5^ U/g, 348 mg/L, and 4.6 mg/g*, P* < 0.01, 0.001 and 0.001, respectively, for each comparison), the high feeding rate (1.9 × 10^5^ U/g, 366 mg/L, and 3.8 mg/g) (*P* < 0.001 for each comparison), and for the control (2.2 × 10^5^ U/g, 383 mg/L, and 3.9 mg/g) (*P* < 0.001 for each comparison) (Fig. 2c; Table 1). Under biomass*-stat* strategy, a near zero specific growth rate was observed when the middle methanol feeding rate was applied. The average specific production rates among the three feeding rates were 0.036 (low feeding), 0.024 (middle feeding) and 0.019 (high feeding) mg/g/h, respectively (Table 1).

### Fermentation Using Reduced Induction DO Value

**Figure 3:**
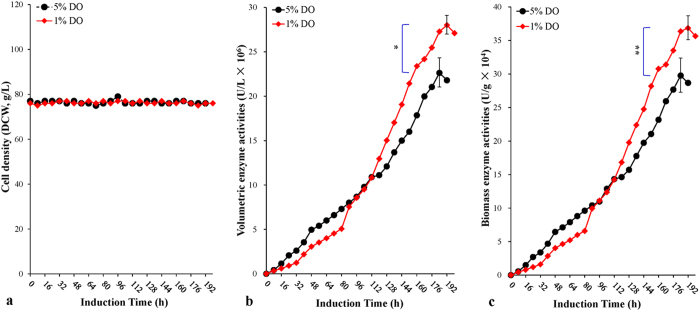
Time-course profiles of the two different induction DO values. (**a**) Cell density profiles (DCW, g/L). (**b**) Volumetric enzyme activity profiles (U/L). (**c**) Biomass enzyme activity profiles (U/g). *Note*, 1% DO (filled diamond); 5% DO (filled cycle). Data are means ± SD of three parallel measurements (*P < 0.05, **P < 0.01, ***P < 0.001).

Our previous work showed that, for the production of the enzyme LXYL-P1-2 by the engineered GS115-3.5K-P1-2^29^, 5% DO during the methanol induction phase was more suitable than 20% DO (recommended by Invitrogen, USA). Using the optimized biomass-*stat*, the induction DO value was further reduced to 1%. Figure 3 shows the time-course profiles of the two different DO values. The biomasses were kept nearly constant at an initial induction value of 75 g/L. Maximum values of volumetric enzyme activity, biomass enzyme activity, volumetric enzyme production and biomass enzyme production at 1% DO reached 2.8 × 10^7^ U/L, 3.7 × 10^5^ U/g, 560 mg/L, and 7.4 mg/g, respectively, at 192 hours, which were higher than those (2.3 × 10^7^ U/L, 3.0 × 10^5^ U/g, 452 mg/L and 6.0 mg/L at 184 hours) at 5% DO (*P* < 0.05, 0.01, 0.001 and 0.001, respectively, for each comparison). However, the peak time at 1% DO was delayed eight hours compared with the latter. The average specific growth rate and average specific production rate at 1% DO were similar to those after the optimization of fermentation using biomass-*stat* strategy (0.025 mg/g/h *vs*. 0.024 mg/g/h, *P* > 0.05).

### Optimization of Initial Induction Biomass

**Figure 4:**
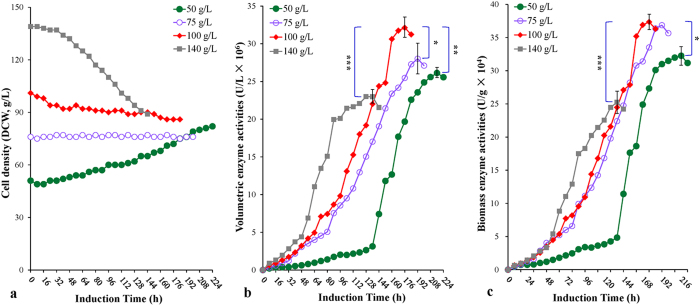
Effects of the initial induction biomass on the HCDF. (**a**) Cell density profiles (DCW). (**b**) Volumetric enzyme activity profiles (U/L). (**c**) Biomass enzyme activity profiles (U/g). Data are means ± SD of three parallel measurements (*P < 0.05, **P < 0.01, ***P < 0.001). *Note*, the initial induction biomasses (g/L) were: 50 (filled cycle), 75 (open cycle), 100 (filled diamond) and 140 (filled square), respectively.

The initial induction biomass was optimized again in the 10-L scale fermenter. The HCDF conditions were as follows: air pressure 0.10 ± 0.05 MPa; methanol feeding rate 0.035 mL/g/h (biomass-*stat* strategy); induction DO value 1%. The initial induction biomasses that were tested included 50 g/L, 75 g/L, 100 g/L and 140 g/L (Fig. 4a). For the initial induction biomass of 100 g/L, maximum values of volumetric enzyme activity, biomass enzyme activity, volumetric enzyme production and biomass enzyme production were 3.2 × 10^7^ U/L, 3.7 × 10^5^ U/g, 642 mg/L and 7.4 mg/g, respectively, much higher than those of the initial induction biomasses of 50 g/L (*P* < 0.01, 0.05, 0.001 and 0.001, respectively, for each comparison) and 140 g/L (*P* < 0.001, for each comparison). Moreover, the peak time and the biomass at the peak time were at 176 hours and 85 g, respectively, which were the shortest peak time and the middle level of biomass among the three initial induction biomasses (Table 1). As a control, the initial biomass of 75 g/L exhibited a lower maximum volumetric activity (2.8 × 10^7^ U/L) and volumetric enzyme production (560 mg/L) compared with the initial biomass of 100 g/L (*P* < 0.05 and 0.01, for each comparison), but showed the same maximum biomass enzyme activity, maximum biomass enzyme production and the same average specific production rate compared with the latter (Table 1). However, the enzyme activity peak time and the biomass at the peak time of the former were at 192 hours and 75 g, respectively, which were the longest peak time and the lowest biomass among the four initial induction biomasses (*P* < 0.001 for each comparison) (Fig. 4a–c; Table 1). Thus, the optimal initial induction biomass was determined to be 100 g/L (DCW). Additionally, the average specific production rates were 0.014 (initial biomass 50 g/L), 0.025 (initial biomass 75 g/L), 0.025 (initial biomass 100 g/L) and 0.025 (initial biomass 140 g/L) mg/g/h, respectively.

The total optimized results are summarized in Table 1.

### Scaling Up HCDF from Pilot Scale to Demonstration/Commercial Scale

**Figure 5:**
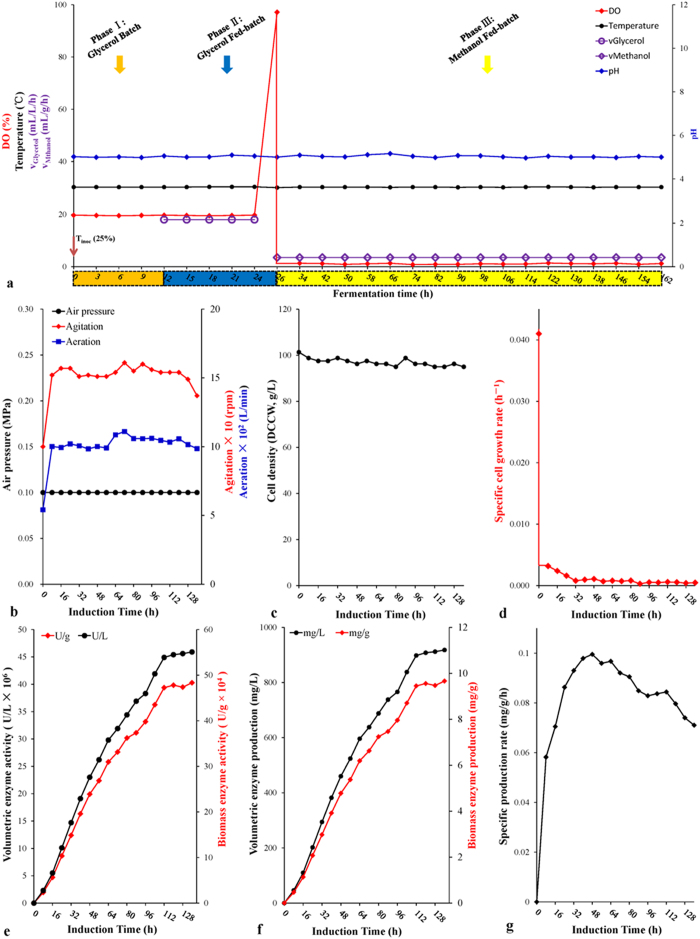
Fed-batch HCDF characterization of 1,000-L scale. (**a**) Profiles of glycerol feeding rate, methanol feeding rate, DO level, temperature and pH during the whole process. (**b**) Air pressure, agitation and aeration profiles. (**c**) Cell density (DCW) profile. (**d**) Specific growth rate (μ, h^−1^) profile. (**e**) Volumetric enzyme activity (U/L) and biomass enzyme activity (U/g) profiles. (**f**) Volumetric enzyme production (g/L) and biomass enzyme production (mg/g) profiles. (**g**) Specific production rate (q_P_) profile. Data are means of three parallel measurements.

**Figure 6:**
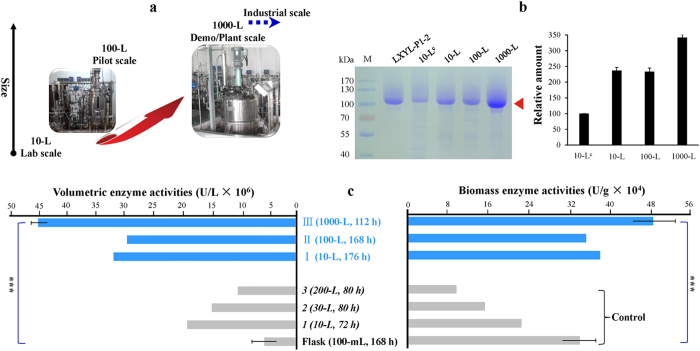
Scaling-up HCDF of *P. pastoris* under 10-L, 100-L and 1,000-L scales. (**a**) Multi-size fermenters scaling-up graphics. (**b**) SDS-PAGE analysis. The arrow indicates the band of the recombinant enzyme. The relative protein amount (arbitrary unit) of each sample was quantified with Quantity One Software. The values are indicated as means ± SD (*n* = 3) of independent assays. M, the protein molecular marker; LXYL-P1-2, the purified recombinant enzyme (glycosylated protein) prepared by gel column choromatography (HPLC) in our lab; 10-L^C^, the sample before optimization (Np-Ox, as a control); 10-L, 100-L and 1,000-L, the samples after optimization. (**c**) Summary of the optimization and scaling-up processes with the controls of the flask fermentation (100-mL), the 10-L scale HCDF (Np-Ox), the 30-L scale HCDF (Np-Ox), and the 200-L scale HCDF (Np-Ox) before optimization, showing the volumetric and biomass enzyme activities at the peak time (except the 112 h in the 1,000-L scale). Error bars show means ± SD of triplicate independent assays.

Using the optimized conditions [air pressure 0.10 ± 0.05 MPa; methanol feeding rate 0.035 mL/g/h (biomass*-stat* strategy); induction DO value 1%; initial induction biomass 100 g/L], the fermentation was scaled up from the pilot scales of 10-L and 100-L to the demonstration/commercial scale of 1,000-L (Figs 5 and 6; Table 2). The HCDF of the 100-L scale shows that the biomass fell slightly from the initial 100 g/L to 85 g/L DCW at the induction time of 40 hours, and then maintained this level. Maximum values of volumetric enzyme activity, biomass enzyme activity, volumetric enzyme production, and biomass enzyme production were 3.0 × 10^7^ U/L, 3.4× 10^5^ U/g, 595 mg/L and 6.8 mg/g, respectively, at the induction time of 168 hours (Table 2), which were slightly lower than those of the 10-L scale (*P >* 0.05 for each comparison). Finally, ~55 L of fermentation broth (initial volume 35 L) were harvested with a total biomass of ~4.7 kg of dry cells. In addition, the average specific production rate returned to the normal level of 0.028 mg/g/h. Profiles of the 1,000-L scale HCDF are exhibited in Fig. 5, in which Fig. 5a shows the changes on DO level, temperature, pH, glycerol feeding and methanol feeding during the whole fermentation process; Fig. 5b–d give the profiles of air pressure, agitation, aeration, cell density and specific growth rate, respectively. With the nearly constant specific cell growth rate of zero, biomass was kept at ~95 g/L during the entire induction phase (Fig. 5c,d). The enzyme activities and enzyme productions of the 1,000-L scale increased more rapidly than those of the 10-L and 100-L scales. At the induction time of 112 hours, volumetric enzyme activity, biomass enzyme activity, volumetric enzyme production and biomass enzyme production of the 1,000-L scale were up to 4.5 × 10^7^ U/L, 4.7 × 10^5^ U/g, 898 mg/L, and 9.4 mg/g, respectively (Fig. 5e,f), which are the best results among the three scales (*P* < 0.001, for each comparison) (Table 2). Furthermore, the biomass enzyme activity was even higher than that of flask fermentation (*P* < 0.001). The exponential increase in enzyme activity of the 1,000-L scale was maintained until 112 hours, and at 136 hours the enzyme activity was still rising. At the induction time of 136 hours, the HCDF of the 1,000-L scale was terminated and ~650 L of fermentation broth (initial volume 400 L) was obtained with the enzyme activities of 4.6 × 10^7^ U/L and 4.8 × 10^5^ U/g (Fig. 5c,d) and a total biomass of ~62 kg of dry cells. The specific production rate increased steadily before 48 hours up to the maximum value of 0.100 mg/g/h and then it continually decreased (Fig. 5g), with the average specific production rate of 0.081 mg/g/h.

The total processes scaling up results (10-L, 100-L to 1,000-L scales) are summarized in Table 2.

The improvement of biomass enzyme production, maximum volumetric enzyme activity and maximum biomass enzyme activity of different scales through step-by-step optimization and scaling up is summarized in Fig. 6b,c, in which Fig. 6b shows the SDS-PAGE result. Samples were from the harvest of each experiment. The maximum values of volumetric and biomass enzyme activities of the three optimized scales surpassed those of flask fermentation, in which the volumetric enzyme activity of the 1,000-L scale was ~7 times higher than that of the flask fermentation while its biomass enzyme activity was also beyond that of the latter (*P* < 0.001). Additionally, the cell density of the fermentation broth harvested from the HCDF of the 1,000-L scale was ~5 times higher than that of the flask fermentation.

## Discussion

*P. pastoris* has become a versatile expression system for scaling up recombinant protein production^6^,^31^,^32^, and a growing number of protein products are hitting the market^33^,^34^. In our previous studies, we cloned the glycoside hydrolase LXYL-P1-2 from *L. edodes*, expressed the enzyme in the *P. pastoris* host and performed the preliminary HCDF of engineered yeast^28^,^29^,^30^. Since the enzyme was produced in non-secreted form, all of the cells were harnessed as the biocatalyst to remove xylosyl residual from 7-*β-*xyloxyl-10-deacetyltaxol (XDT) (and other 7-*β-*xylosyl-taxanes) in order to form 10-deacetyltaxol (DT), which is the semi-synthetic precursor of Taxol. Using freeze-dried yeast cells harvested from HCDF, we performed the bioconversion of 10 L^30^. Since the biomass enzyme activity of HCDF was apparently lower than that of flask fermentation, improving this activity would be beneficial to saving a large amount of production costs. Additionally, a great deal of pure oxygen was consumed during HCDF, but its practical application for large-scale fermentation is limited as it is dangerous to handle and is expensive. Therefore, the elimination of a pure oxygen supplement is an economical approach to HCDF, particularly for large-scale fermentation.

In this paper, we first demonstrated that a pure oxygen supplement could be omitted from HCDF if jar air pressure is increased from the normal 0.05 MPa (super atmospheric pressure) to 0.10±0.05 MPa. In the Ip-Air experiment, average specific production rate of LXYL-P1-2 reached 0.023 mg/g/h, which was similar to that of the Np-Ox experiment (0.022 mg/g/h) (*P* > 0.05). Maximum volumetric and biomass enzyme activities reached 2.1 × 10^7^ U/L and 1.9 × 10^5^ U/g (DCW), corresponding to the maximum volumetric and biomass enzyme productions of 420 mg/L and 3.8 mg/g, respectively. The designated activities and enzyme productions even surpassed those of the Np-Ox experiment (1.5 × 10^7^ U/L, 1.5 × 10^5^ U/g, 294 mg/L and 3.0 mg/L) (*P* < 0.01, 0.05, 0.001 and 0.005, respectively, for each comparison), although the peak time in the Ip-Air experiment was twice as long as that in the Np-Ox experiment (Fig. 1a–c). The additional methanol consumed in the Ip-Air experiment was still cheaper than the pure oxygen consumed in the Np-Ox experiment. Similar approaches have been used in the production of other heterologous proteins of the yeast system, improving biomass production and/or enzyme activity^11^,^13^,^14^,^15^,^16^,^17^,^19^,^21^,^23^. However, to our knowledge, there are no publications that show the use of the Ip-Air condition in demonstration scale fed-batch HCDF for the expression of the foreign protein of *P. pastoris*.

Second, we developed the biomass-*stat* methanol feeding strategy based on the DO-*stat* strategy. Using this new strategy in the Ip-Air experiment with an optimal methanol feeding rate (*v* = 0.035 mL/g/h), maximum enzyme activities and productions were further improved compared with those of the control (*P* < 0.001 for each comparison), arriving at 2.3 × 10^7^ U/L, 3.0 × 10^5^ U/g, 452 mg/L, and 6.0 mg/g (at 184 hours), respectively. Moreover, the biomass enzyme activity was even closer to that of flask fermentation (3.3 × 10^5^ U/g at 168 hours) (*P* > 0.05) while its volumetric enzyme activity was four times higher than the latter. (Figs 2b,c and 6c). Under the biomass-*stat* condition, the average specific production rate was 0.024 mg/g/h (*vs*. 0.022 mg/g/h in the control), and a higher production was achieved.

For methylotrophic yeast in the induction phase, methanol was the sole carbon source and inducer of the *AOX1* promoter, and the feeding strategy directly affected the expression level of the heterologous protein. Reasonable energy distribution and consumption between the protein expression and cell growth was of particular importance. Other methanol feeding strategies, such as DO-*stat*, μ*-stat*, OLFB and MNLFB are non-continuous or interval methanol feeding processes and methanol concentration is non-uniform. In contrast, the main concept of the biomass-*stat* strategy is that biomass is defined and the relationship between biomass and methanol feeding rate is immediately quantified. Meanwhile, the constant methanol feeding rate was adjusted in order to maximize the production of heterologous proteins. It seems that the high expression level of LXYL-P1-2 in *P. pastoris* was a subsequent contribution of the continuous supplement of methanol with non-inhibitory residual. We anticipate that this new strategy will also apply to the production of other heterologous proteins through the HCDF of *P. pastoris*, but only when real time cell density is measured.

Third, in the Ip-Air experiment, we lowered the induction DO value to 1% for the HCDF of *P. pastoris* and biomass-*stat* strategy in order to evaluate the effects of a lower induction DO value on enzyme activity. This was due to the fact that, in our previous work, we found that an induction DO value of 5% was better than an induction DO value of 20%, which is recommended by the protocol of the “*Pichia* Fermentation Process Guidelines” (Invitrogen, USA) for the HCDF of *P. pastoris* in LXYL-P1-2 expression^29^. Indeed, maximum enzyme activities and productions were obviously higher than those of the control (Fig. 3b,c; Table 1) (P < 0.05 for the comparisons of the enzyme activities; *P* < 0.001 for the comparisons of the enzyme productions). Moreover, the maximum biomass enzyme activity (3.7 × 10^5^ U/g) surpassed that of flask fermentation (Figs 3b,c and 6c) (*P* > 0.05).

Similar phenomena have been observed elsewhere^35^,^36^. Another report showed that oxygen can be limited during fed-batch to limit growth and maintain a higher methanol concentration. This, in turn, may further induce the *AOX1* promoter to more efficiently express the heterologous gene^37^. It was also reported that post-translational modifications were reduced in oxygen-limited cultivations^38^, thus improving the purity of products secreted into the medium^37^. Limiting oxygen also reduced cell lysis^37^. However, due to increased cell density and longer induction time, the methanol requirements of these oxygen-limited conditions were higher^24^,^39^,^40^, agreeing with our results (Fig. 3a–c). The benefit of the low DO level was also found in the *P. pastoris* system with other carbon source. Pérez-Martínez *et al.* evaluated the expression of the recombinant *Trichoderma atroviride* endochitinase *ech42* (*rech42*) in *P. pastoris* driven by the constitutive promoter P_*GAP*_ at low (3%) and high (40%) DO tensions, finding that the low DO tension could improve the volumetric production of *Rech42*^36^.

Fourth, we further optimized the initial induction biomass. It was reported that the optimization of pre-induction (initial induction) biomass could increase the expression of GFP on the 3-L scale of fed-batch cultures^41^. In our previous work, we determined the optimum initial induction biomass to be 300 g/L (WCW, equivalent to 75 g/L DCW) in the Np-Ox experiment^29^. In the Ip-Air experiment and with an induction DO value of 1%, as well as the biomass-*stat* strategy, the optimum initial induction biomass was determined to be 100 g/L (DCW). With this initial induction biomass, maximum volumetric and biomass enzyme activities reached 3.2 × 10^7^ U/L and 3.7 × 10^5^ U/g (at 176 hours), respectively, in which the maximum volumetric enzyme activity surpassed that of the initial induction biomass of 75 g/L (2.8 × 10^7^ U/L at 192 hours) (Fig. 4a,b; Table 1). The former also produced more biomass in addition to a peak time.

Finally, using the optimized conditions mentioned above, we scaled up the HCDF experiment from the pilot scales of 10-L and 100-L to the demonstration/commercial scale of 1,000-L. The experimental results were reproducible on both the low and higher scales, but the best results were obtained on the 1,000-L scale as volumetric enzyme activity, biomass enzyme activity, volumetric enzyme production and biomass enzyme production were no less than 4.5 × 10^7^ U/L, 4.7 × 10^5^ U/g, 898 g/L and 9.4 mg/g (Fig. 5e,f), respectively, with average specific production rate of 0.081 mg/g/h and maximum specific production rate of 0.100 mg/g/h (Fig. 5g; Table 2). Not only were enzyme activity, production and biomasses well beyond the flask fermentation levels (*P* < 0.001), but the fermentation period was also apparently shorter (*P* < 0.001). Moreover, the trend of the activity-time curve was similar to that of flask fermentation as both volumetric and biomass enzyme activities continued to increase slowly or remained at a non-reduced level after 112 hours. Furthermore, due to the sound aeration conditions, we found that the conditions of the Ip-Air experiment were particularly suitable for larger volume fermentations.

The technology stage gates for the development of processing technology are generally divided into four scales: 1) bench or lab scale (reactor volumes < 1,000 mL); 2) pilot (plant) scale (reactor volumes range from 1 L to 100 L); 3) demonstration (plant) scale (reactor volumes fall within the range of 100 L to 1,000 L); and 4) commercial scale (reactor volumes ≥1,000 L). The bench or lab scale is an important early-stage tool, the pilot scale provides the first window into continuous processing and the demonstration scale more closely resembles the commercial scale in terms of equipment and processing flow sheets. It is also the final technological hurdle in the decision to go commercial^42^. For the recombinant *P. pastoris* GS115-3.5K-P1-2, the 1,000-L scale has possessed commercial implications.

The scaling up of the microbial fermentation process is a vital step that bridges lab research and commercialization. For the *P. pastoris* system, although significant attention has been paid to the optimization of HCDF, including DO levels and methanol feeding strategies, few publications focus on scaling up to large volumes, especially volumes of more than 100-L^43^,^44^. Baumgartner *et al.* investigated the production of recombinant phytohemagglutinin E-form (PHA-E of *Phaseolus vulgaris* origin) in *P. pastoris*. The yield of secreted PHA-E was approximately 100 mg/L from HCDF on the 2-L and 200-L scales^44^. They also produced agglutinin from *Galanthus nivalis* (GNA) on the 200-L scale, and the GNA was secreted at approximately 200 mg/L in the medium^43^. In order to produce an ice-binding protein (rLeIBP) from a fed-batch culture of *P. pastoris*, Lee *et al.* developed a process to scale up HCDF. The recombinant protein yields on the 7-L and 700-L scales were ∼272 mg/L and 300 mg/L, respectively^32^. However, all of these scaling-up processes were conducted with standard air pressure and oxygen enriched aeration was normally required. To our knowledge, for stirred tank bioreactors, the reproducible scaling up of the HCDF of *P. pastoris* presented here represents the largest scale ever reported in literature.

In order to realize the industrial production of highly active recombinant yeast cells and mass production of DT (the semi-synthetic precursor of Taxol) through the bioconversion of the naturally redundant XDT, we optimized the process of the HCDF of a recombinant *P. pastoris* harboring the specific *β*-xylosidase gene. The work included setting up the conditions of the Ip-Air experiment, developing a new biomass-*stat* methanol feeding strategy, reducing the induction DO value to 1% and using an initial induction biomass of 100 g/L (DCW). Under optimum conditions, maximum biomass LXYL-P1-2 activity increased gradually until finally surpassing that of flask fermentation. Additionally, HCDF yielded several times more biomass. High biomass enzyme activity was reproduced and scaled up from the 10-L scale through the 100-L to 1,000-L scales, and the best results were obtained on the 1,000-L scale. Our present study lays a solid foundation for the commercial preparation of DT from the substrate XDT, using recombinant yeast for the industrial semi-synthesis of Taxol. It also provides a successful paradigm for scaling up the HCDF of *P. pastoris* to the demonstration/commercial scale. Our new biomass-*stat* methanol feeding strategy can also be applied to the production of other heterologous proteins from the HCDF of *P. pastoris*.

## Methods

### Strain, Media and Fermenters

The engineered strain GS115-3.5K-P1-2 was constructed in our laboratory by transforming the host strain *P. Pastoris* GS115 (Mut^+^) with the recombinant plasmid pPIC3.5K-LXYL-P1-2 (intracellular expression) harboring the sequence encoding LXYL-P1-2^28^, and cryopreserved at −80 °C prior to use. A yeast extract peptone dextrose (YPD) agar plate containing (*w/v*) 1% yeast extract, 2% peptone, 2% glucose and 1.5% agar was used to activate the GS115-3.5K-LXYL-P1-2 strain. Additionally, a BMGY medium containing (per liter) 10 g of yeast extract, 20 g of peptone, 100 mm of potassium phosphate buffer pH6, 13.4 g of yeast nitrogen base without amino acids, 400 μg of biotin and 10 mL of glycerol was used for a seed culture in shaking flasks. An FM22 medium containing (per liter) 42.9 g of KH_2_PO_4_, 5 g of (NH_4_)_2_SO_4_, 1 g of CaSO_4_·2H_2_O, 14.3 g of K_2_SO_4_, 11.7 g of MgSO_4_·7H_2_O and 40 g of glycerol was used for HCDF and 4.35 mL of *Pichia* trace minerals 4 (PTM4) was added per liter of the initial FM22 medium. Finally, a PTM4 salts solution containing (per liter) 2 g of CuSO_4_·5H_2_O, 0.08 g of NaI, 3 g of MnSO_4_·H_2_O, 0.2 g of Na_2_MoO_4_·2H_2_O, 0.02 g of H_3_BO_3_, 0.5 g of CaSO_4_·2H_2_O, 0.5 g of CoCl_2_, 7 g of ZnCl_2_, 22 g of FeSO_4_·7H_2_O, 0.2 g of biotin and 1mL of H_2_SO_4_ was prepared. Other solutions, including a 50% glycerol, methanol and antifoam regent, were the same as those in our previous published literature^29^,^30^. The fermenters were as follows: 1) 10-L size, 10S type, Shanghai Gaoji Bioengineering Co., Ltd., Shanghai, China; 2) 30-L size, 30S type, Yangzhong Weikete Bioengineering Equipment Co., Ltd., Zhenjiang, China; 3) 100-L size, 100S type, Shanghai Gaoji Bioengineering Co., Ltd., Shanghai, China; 4) 1,000-L size, 30/200/1,000-L type, Zhenjiang Dongfang Bioengineering and Biotechnology Co., Ltd., Jiangsu, China.

### Biomass Measurement and Enzyme Assay

The cell density measurement and enzyme assay methods we used were similar to those in our previous work. Briefly, samples were taken during the time course of fermentation and aliquots of 1 mL were centrifuged at 12,000 rpm for 3 min. The cell pellet was washed three times with distilled water and weighed. Then, aliquots of the cell pellet were freeze-dried to a constant weight. Biomass was gravimetrically expressed as the dry cell weight (DCW). The chromogenic substrate *p*-Nitrophenyl-*β*-D-xylopyranoside (PNP-Xyl, Sigma, USA) was used to monitor enzyme activity. This activity was then evaluated by calculating both U/L (volumetric enzyme activity) and U/g (biomass enzyme activity) as described in the literature^29^. One unit of enzyme activity was defined as the amount of enzyme required to release one nM *p*-nitrophenol from PNP-Xyl per minute at 50 °C and pH5.

### Fed-batch HCDF

#### Shaking flask cultivation

The GS115-3.5K-LXYL-P1-2 strain from the glycerol stock culture at −80 °C was streaked onto an YPD agar plate containing 4 mg/mL of G418. The plate was then incubated at 30 °C for ~3 days. A single colony was inoculated into 500 mL shaking flasks, each containing 100 mL of the BMGY medium. The flasks were incubated at 30 °C for 20 hours in a shaking incubator (220 rpm).

#### Glycerol batch fermentation (S1)

The procedure previously established in our lab^29^, which was based on the protocol described in the manufacturer’s handbook (Invitrogen, USA), was followed by HCDF of different scales. For the 10-L scale, the initial working volume and inoculum concentration were 3.5 L and 10% (*v/v*), respectively. The initial working volume and inoculum concentration for the 30-L scale were 10.5 L and 5% (*v/v*), respectively. With regard to the 100-L scale, a 350 mL seed culture from the shaking flasks was used as inoculum for a 10-L fermenter containing 3.5 L of the FM22 medium plus 4.35 mL/L of PTM4 salts. When the second seed culture reached a cell density of ~28.5 g/L DCW, 3.5 L of the culture was directly added to 35 L of the FM22 medium in a 100-L fermenter. The 1,000-L scale was also conducted using the second seed culture and, 1 L of the first seed culture from the shaking flasks was inoculated in a 200-L fermenter containing 100 L of the FM22 medium plus 4.35 mL/L of PTM4 salts. When the second seed culture reached a cell density of ~25 g/L DCW, 100 L of the culture were directly added to 400 L of the FM22 medium in a 1,000-L fermenter. Following this, the glycerol batch phase was initiated and the fermentation conditions were maintained at 30 °C, pH5, 0.10 ± 0.05 MPa and DO of 20% saturation by controlling the aeration, agitation and air pressure. Additionally, an ammonia solution (28%), which served as the nitrogen source at the same time, was used to maintain the medium pH5. An anti-foam agent (JXPF-1208) was also used to prevent foaming.

#### Glycerol fed-batch fermentation (S2)

After the initial glycerol in the FM22 medium was consumed, we switched to a glycerol fed-batch culture with a feeding of 50% glycerol (*v/v*), containing 1.2% PTM4 salts (*v/v*), at a constant rate of 18 mL/L/h. All fermentation conditions, including temperature, pH, air pressure, DO and anti-foam agent were the same as those in S1.

#### Methanol fed-batch fermentation (S3)

Glycerol feeding was kept until the biomass (DCW, g/L) reached the required level, then the feeding was terminated but the cultivation continued. Indicated by the sudden rise of DO to ≥90%, methanol induction was initiated by adding a methanol solution (100%) containing 12 mL/L of PTM4 salts based on our previous reports^29^,^30^. Unless otherwise indicated, 5% DO and 75 g/L (DCW) of the initial induction biomass were used for the entire induction period. Every three hours, samples were taken from the fermenter for biomass analysis, and every eight hours for both biomass analysis and enzyme assay with the chromogenic substrate PNP-Xyl. This study developed and used a new methanol feeding strategy. We designed the strategy as biomass-*stat*, meaning biomass was maintained at a nearly constant level during the methanol induction phase. Afterwards, the methanol feeding rate (*v*) was determined according to the equation *v* = *V/m/t*; *V* is the feeding volume of methanol (mL), *m* is the biomass of DCW (g/L) and *t* is the feeding time (h).

### HCDF Optimization and Scaling Up

HCDF optimization was carried out on the 10-L and 30-L scales. First, following the DO sustaining model (DO-*stat* strategy), pure oxygen was supplemented during fermentation to maintain proper DO values (20% for S1 and S2, 5% for S3 and air pressure 0.05 MPa). Then, the pure oxygen was eliminated by increasing the jar air pressure from 0.05 Mpa to 0.10 ± 0.05 MPa (super atmospheric pressure). Subsequently, the methanol feeding rate was further optimized according to the biomass-*stat* strategy (no pure oxygen supplement). Three different methanol feeding rates (*v*), namely, a low level rate (0.025 mL/g/h), a middle level rate (0.035 mL/g/h) and a high level rate (0.050 mL/g/h), were compared. The DO value in the methanol induction phase was then optimized from 5% to 1%. Finally, the initial induction biomass was further optimized among the 50 g/L, 75 g/L, 100 g/L and 140 g/L (DCW) cell densities.

The optimized HCDF was scaled up from the 10-L scale to the 100-L and 1,000-L scales. The fermentation conditions were as follows: temperature 28 ~ 30 °C; pH5; air pressure 0.10 ± 0.05 MPa (without addition of pure oxygen); methanol feeding rate *v* = 0.035 mL/g/h (biomass-*stat*); induction DO 1%; initial induction biomass 100 g/L (DCW). For HCDF on the 10-L, 100-L and 1,000-L scales, the aeration was altered within the ranges of 2-10 L/min, 20-100 L/min and 200-1,000 L/min, respectively, and the agitation was adjusted within the ranges of 100-680 rpm, 50-400 rpm and 20-280 rpm, respectively.

### Cell Harvesting

At the end of fermentation, cells were collected and washed at least three times using distilled water through centrifugation. The fermentation broths from the HCDF of the 10-L and 100-L scales were collected and run through a large-capacity centrifuge at ~6,000 rpm for 15 minutes (Sigma 8K, Sigma, St. Louis, MO, USA). The fermentation broth from the HCDF of the 1,000-L scale was run through a disk centrifuge at 9,300 rpm (Clara 20, Alfa Laval Separation AB, Lund of Sweden). The washed cell pellet was freeze-dried and stored at −20 °C prior to use.

### Enzyme Isolation and SDS-PAGE Analysis

The recombinant enzyme was isolated based on our previous report^45^. Briefly, 5 g freeze-dried yeast cells were suspended in 50 mL Tris-HCl buffer (20 mM, pH 8.0) and subjected to 10 cycles of high-pressure cell disruption (APV-2000, SPX Corporation, USA) at 1,200 bar, 4 °C. The supernatant was obtained by centrifugation at 15,000 rpm and 4 °C for 30 min. After gradient elution of different concentration of imidazole (buffer A: 20 mM, pH8.0; buffer B: 60 mM, pH8.5; buffer C: 200 mM, pH8.0) by nickel bonded affinity chromatography, samples eluted from buffer B were collected and concentrated (UFC903096 30KD, Millipore, USA). The concentrated samples were then subjected to SDS-PAGE analysis by means of our previous work^28^. Broadly speaking, samples of 10 μL were mixed with 2.5 μL of sample buffer (5 × sample buffer, Invitrogen) and incubated for 5 min at 100 °C. Then, SDS-PAGE was performed using a 5% stacking gel and a 12% separating gel on a vertical mini gel apparatus (Bio-Radmini-2D, Bio-Rad, USA), as described by Laemmli^46^. Protein molecular weight marker was purchased from NEB (P7702, New England). The purified recombinant LXYL-P1-2 was prepared previously by our lab and was used as the control. Gels were stained with Coomassie Brilliant Blue R-250 (Sigma Chemical, St. Louis, MO). The protein bands on SDS-PAGE gels were quantified with arbitrary unit by Quantity One software version 4.5.0 (Bio-Rad).

### Other Calculations and Statistical Analysis

Calculations of volumetric enzyme production (mg/L), biomass enzyme production (mg/g) and specific enzyme production rate (mg/g/h) were based on the specific activity of the purified LXYL-P1-2, which is 5 × 10^4^ U/mg. The parameters were calculated by the following equations (Eq.), namely, specific (cell) growth rate (μ) = 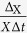 (Eq. 1); volumetric enzyme production (mg/L) = volumetric enzyme activity (U/L) ÷ (5 × 10^4^ U/mg) (Eq. 2); biomass enzyme production (mg/g) = biomass enzyme activity (U/g) ÷ (5 × 10^4^ U/mg) (Eq. 3); specific (enzyme) production rate (q_P_ )= volumetric enzyme production (mg/L) ÷ (XΔt)
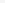
(Eq. 4), where X and t are the cell density (or biomass) (g/L, DCW) and time (h), respectively. For each data, replicates from three parallel measurements or independent assays were measured and the mean ± standard error (SD) was calculated. Student’s *t*-test in SPSS 17.0 (SPSS Inc.) was used for two-group comparisons. *P* < 0.05 was considered statistically significant.

## Additional Information

**How to cite this article**: Liu, W.-C. *et al.* Scaling-up Fermentation of *Pichia pastoris* to demonstration-scale using new methanol-feeding strategy and increased air pressure instead of pure oxygen supplement. *Sci. Rep.* **6**, 18439; doi: 10.1038/srep18439 (2016).

## Figures and Tables

**Table 1:** Summary of the fed-batch HCDF optimization process of *P. pastoris*.

Contents		Specific growthrates (μ, h^−1^)^a^	VolumetricEnzymeactivities(U/L × 10^7^)^b^	Biomassenzymeactivities(U/g × 10^5^)^b^	Volumetricenzymeproduction(mg/L × 10^2^)^b^	Biomassenzymeproduction(mg/g)^b^	Specificproductionrates(q_P_, mg/g/h)^a^	Peaktime (h)	Biomasses(g/L)^b^
Air pressure mode	0.05 MPa, Np-Ox (control)	0.0034 ± 0.0014	1.5 ± 0.3	1.5 ± 0.2	2.9 ± 0.2	3.0 ± 0.4	0.022 ± 0.001	80	97.5
	0.10 ± 0.05 MPa, Ip-Air	0.0040 ± 0.0015	2.1 ± 0.2	1.9 ± 0.1	4.2 ± 0.3	3.8 ± 0.2	0.023 ± 0.002	156	107.5
Biomass-*stat*	0.025 mL/g/h	~0	1.7 ± 0.2	2.3 ± 0.4	3.5 ± 0.1	4.6 ± 0.1	0.036 ± 0.002	152	75
	0.035 mL/g/h	~0	2.3 ± 0.2	3.0 ± 0.3	4.5 ± 0.1	6.0 ± 0.3	0.024 ± 0.003	184	75
	0.050 mL/g/h	0.0032 ± 0.0012	1.8 ± 0.1	1.9 ± 0.1	3.7 ± 0.2	3.8 ± 0.4	0.019 ± 0.002	144	95
Induction DO level	1%	~0	2.8 ± 0.4	3.7 ± 0.3	5.6 ± 0.1	7.4 ± 0.5	0.025 ± 0.004	192	75
	5%	~0	2.3 ± 0.2	3.0 ± 0.3	4.5 ± 0.1	6.0 ± 0.4	0.024 ± 0.003	184	75
Initial induction biomass	50 g/L	0.0037 ± 0.0013	2.6 ± 0.1	3.2 ± 0.2	5.2 ± 0.3	6.4 ± 0.3	0.014 ± 0.003	216	80
	75 g/L	~0	2.8 ± 0.4	3.7 ± 0.3	5.6 ± 0.1	7.4 ± 0.5	0.025 ± 0.004	192	75
	100 g/L	~0	3.2 ± 0.2	3.7 ± 0.1	6.4 ± 0.1	7.4 ± 0.3	0.025 ± 0.001	176	85
	140 g/L	0.0073 ± 0.0016	2.3 ± 0.3	2.5 ± 0.3	4.6 ± 0.2	5.0 ± 0.2	0.025 ± 0.002	136	90

^a^Average values of all sampling points before peak time (means ± SD), three parallel measurements for each sampling point.

^b^Values (means ± SD, three parallel measurements) at peak time.

**Table 2:** Comparison of different fed-batch HCDF scales under optimized conditions.

HCDF scales	Specificgrowthrates (μ)(h^−1^)^a^	Volumetricenzymeactivities(U/L × 10^7^)^b^	Biomassenzymeactivities(U/g × 10^5^)^b^	Volumetricenzymeproduction(mg/L × 10^2^)^b^	Biomassenzymeproduction(mg/g)^b^	Specificproductionrates (q_P_)(mg/g/h)^a^	Peaktime (h)^c^	Biomasses(g/L)^b^
10-L	~0	3.2 ± 0.2	3.7 ± 0.1	6.4 ± 0.1	7.4 ± 0.3	0.025 ± 0.001	176	85
100-L	~0	3.0 ± 0.4	3.4 ± 0.3	6.0 ± 0.2	6.8 ± 0.4	0.028 ± 0.004	168	85
1,000-L	~0	4.5 ± 0.3	4.7 ± 0.5	9.0 ± 0.3	9.4 ± 0.3	0.081 ± 0.003	112	95

^a^Average values of all sampling points before peak time (means ± SD), three parallel measurements for each sampling point.

^b^Values (means ± SD, three parallel measurements) at peak time or at 112 hours for 1,000-L scale.

^c^Except that of 1,000-L scale.
